# Background and features of the WHO hand hygiene self-assessment framework

**DOI:** 10.1186/1753-6561-5-S6-O68

**Published:** 2011-06-29

**Authors:** B Allegranzi, A Stewardson, L Grayson, E Larson, A Voss, C Kilpatrick, D Pittet

**Affiliations:** 1 World Health Organization, Geneva, Switzerland; 2 University of Geneva Hospitals, Geneva, Switzerland; 3 Hand Hygiene Task Force, World Health Organization, Geneva, Switzerland

## Introduction / objectives

To develop a tool for self-assessment of HH resources, practice and promotion in healthcare facilities (HCF), based on the key components of the WHO Multimodal Hand Hygiene (HH) Improvement Strategy (MHHIS).

## Methods

A task force (TF) of HH experts developed the tool and identified the following desired features: 1) to reflect the 5 components of the WHO MHHIS; 2) to include measurable indicators based on scientific evidence and expert consensus; 3) to be in the format of a user-friendly questionnaire; 4) to be associated with a score; 5) to be usable repetitively over time. Tool development steps were: 1) identification of indicators, score and format; 2) usability and reliability pilot testing; 3) review and finalization.

## Results

The tool was named HH Self-Assessment Framework (HHSAF) and structured in 5 sections corresponding to the WHO MHHIS components (system change; training and education; evaluation and feedback; reminders in the workplace; institutional safety climate for HH). 27 indicators were included and points assigned according to available evidence and importance attributed by experts. WHO implementation tools suited to the improvement of each indicator were referred to within the HHSAF. A score of maximum 100 points was established per each HHSAF section. According to the overall score, 4 HH situation levels were identified: inadequate; basic; intermediate; advanced. Additional leadership criteria for HH reference centres were included. The draft was finalized according to results of usability and reliability tests.

## Conclusion

Through a thorough development process, all desired features identified for an optimal HH self-assessment tool at HCF level were successfully fed into the HHSAF.

## Disclosure of interest

None declared.

